# Usefulness of Cerebral Oximetry in TBI by NIRS

**DOI:** 10.3390/jcm10132938

**Published:** 2021-06-30

**Authors:** Małgorzata Barud, Wojciech Dabrowski, Dorota Siwicka-Gieroba, Chiara Robba, Magdalena Bielacz, Rafael Badenes

**Affiliations:** 1 Department of Anaesthesiology and Intensive Therapy, Medical University of Lublin, 20-954 Lublin, Poland; w.dabrowski5@yahoo.com (W.D.); dsiw@wp.pl (D.S.-G.); 2 Department of Anaesthesia and Intensive Care, Policlinico San Martino, 16100 Genova, Italy; kiarobba@gmail.com; 3 Institute of Tourism and Recreation, State Vocational College of Szymon Szymonowicz, 22-400 Zamosc, Poland; magda.bielacz@gmail.com; 4 Department of Anaesthesiology and Intensive Care, Hospital Clìnico Universitario de Valencia, University of Valencia, 46010 Valencia, Spain; rafaelbadenes@gmail.com

**Keywords:** cerebral oximetry, near-infrared spectroscopy, traumatic brain injury, cerebrovascular autoregulation, intracranial pressure

## Abstract

Measurement of cerebral oximetry by near-infrared spectroscopy provides continuous and non-invasive information about the oxygen saturation of haemoglobin in the central nervous system. This is especially important in the case of patients with traumatic brain injuries. Monitoring of cerebral oximetry in these patients could allow for the diagnosis of inadequate cerebral oxygenation caused by disturbances in cerebral blood flow. It could enable identification of episodes of hypoxia and cerebral ischemia. Continuous bedside measurement could facilitate the rapid diagnosis of intracranial bleeding or cerebrovascular autoregulation disorders and accelerate the implementation of treatment. However, it should be remembered that the method of monitoring cerebral oximetry by means of near-infrared spectroscopy also has its numerous limitations, resulting mainly from its physical properties. This paper summarizes the usefulness of monitoring cerebral oximetry by near-infrared spectroscopy in patients with traumatic brain injury, taking into account the advantages and the disadvantages of this technique.

## 1. Introduction

Traumatic brain injuries (TBIs) are some of the main causes of mortality in patients injured as a result of traffic accidents, falls from a height, battery, or firearm assault. TBIs are classified in various ways. Depending on the severity of the injury, they are divided into mild, moderate, and severe injuries. Depending on the mechanism of injury, they are classified into focal injuries and primary diffuse brain injuries. They can be isolated or part of a multi-organ trauma. TBI leads to primary brain injury.

Treatment of patients with TBI is primarily focused on preventing secondary brain injury. For many years, the standard of care for patients with TBI has been the monitoring of intracranial pressure (ICP) and cerebral perfusion pressure (CPP), estimated as the difference between ICP and mean arterial pressure (MAP) [1]. Continuous EEG monitoring is also increasingly used. However, these methods do not provide direct information on hypoxia and ischemia or the metabolic needs of the brain tissue. Hence, there is a need to use a method that would allow the assessment of cerebral oxygenation.

For almost 30 years now, a method of non-invasive monitoring of cerebral oximetry using near-infrared spectroscopy (NIRS) has been available. This technique was initially used in neonatal intensive care [2] and cardiac surgery [3], and has since been increasingly employed in adult intensive care.

## 2. Near-Infrared Spectroscopy (NIRS)

Cerebral oximetry measurements are performed using near-infrared spectroscopy (NIRS). It is a continuous, non-invasive method that enables monitoring of regional cerebral oxygen saturation. Normal NIRS readings represent a balance between oxygen supply and consumption in peripheral tissues.

The technique was first described by Jobsis in 1977 [4]. Under physiological conditions, tissues are relatively translucent to near-infrared light with wavelengths of 700–1000 nm, and the absorption of light by tissues is low. As a result, light can penetrate to a depth of up to 8 cm and still be completely detectable. [5]. Near-infrared light beams can penetrate bones, which is essential for transcranial monitoring of cerebral oximetry.

Cerebral oximetry measurements are cheap and easy to take. Special sensors are applied to the scalp which allow one to obtain measurements mainly from the external parts of the brain. For practical reasons, the sensors are commonly attached to the scalp overlying the frontal lobe, but measurements can be made from above any brain lobe. Each probe has a near-infrared light generator (diode or laser) and a proximal and distal light detector. Mixed arterial and venous blood in the brain and external tissues is measured. The arterial to venous blood ratio in the brain is 15:85 [6]. Hemoglobin oxygenation in brain vessels is estimated using percutaneous measurements of the amount of light absorbed by hemoglobin in the cerebral cortex.

The light transmitted through biological tissues is reflected, absorbed and scattered. Light reflection is determined by the angle between the light beam and the tissue surface. The absorption of light by tissues, causing the attenuation of light, depends on the chromophores—oxyhemoglobin, deoxyhaemoglobin and cytochrome oxidase. NIRS uses the different absorption properties of oxyhemoglobin, deoxyhaemoglobin and cytochrome oxidase to quantify their concentrations in tissues [7]. The relationship between the concentration of chromophore (*c*), its extinction coefficient (*α*), distance traveled by light in tissues (*d*), and the ratio of the incident light intensity (*I*_0_) to the intensity of transmitted light (*I*) is determined by the Beer-Lambert equation [5,8]:

(1) $$A=\log\left(I_{0}/I\right)=\alpha \times c\times d$$

Most photons in tissues are scattered, so the path traveled by photons can be much longer than the direct distance between the optodes. Most of the total attenuation of an infrared light beam is due to scattering, and only a small portion is absorbed. That is why the differential pathlength factor (DPF) and factor G, which depends on light losses other than attenuation, need to be added to the above equation [5,9]:

(2) $$A=\log\left(I_{0}/I\right)=\alpha \times c\times d\times DPF+G$$

NIRS measurements in adults can be performed with the emitter and detectors placed on the same side of the head, providing “regional” information on brain oxygenation as only a small volume of brain tissue between the optodes is examined. In this method, the light travels along an arc with a tissue penetration depth of approximately half the distance between the emitter and the detectors. It is recommended that the distance between the optodes be not less than 2.5 to 3 cm, because the shorter the distance between the optodes, the more strongly the extracerebral tissues attenuate the light [5].

The first NIRS devices used two or three wavelengths and were mainly trend monitors. Nowadays, a four-wavelength generation of NIRS monitors are available, which provide reliable real-time regional cerebral oxygen saturation readings. NIRS devices can use three different detection modes: continuous wave (CW), frequency domain (FD), and time domain (TD). CW monitors are the most popular and the simplest devices. They measure the attenuation of incident light. FD monitors use high-frequency modulation to measure the phase and intensity of the signals generated. This technique allows a more quantitative assessment of the optical properties of tissues. TD monitors measure the time of flight of photons and are the most expensive of all NIRS devices [10].

## 3. Usefulness of Monitoring Cerebral Oximetry in TBI Patients by NIRS

### 3.1. Identification of Episodes of Impaired Cerebral Oxygenation

Adequate cerebral oxygenation is essential in treating patients with TBI. There exist invasive measurement methods, such as the measurement of jugular bulb oxygen saturation (SjvO2) or the measurement of brain tissue oxygen pressure (PbtO2). However, cerebral oximeters using NIRS are more and more frequently employed to obtain non-invasive measurements of cerebral oxygenation.

In a study by Esnault et al., cerebral oximetry (rScO2) values obtained by NIRS were compared with the results of an invasive measurement of brain tissue oxygen pressure. A PbtO2 probe was inserted into the frontal lobe of the cerebral hemisphere with more severe tissue injury/damage. The rScO2 sensor, on the other hand, was placed on the skin in the frontal area ipsilateral to the PbtO2 probe. The authors found no correlation between the readings obtained with NIRS and the tissue oxygen pressure probe. Cerebral oximetry allowed one to identify only about 15% of the ischemic episodes detected by the PbtO2 measurement. When one of the subjects developed brain death, NIRS continued to show normal rScO2 values, while PbtO2 dropped to 0 mmHg. Esnault et al. concluded that rScO2 measurement could not be used interchangeably with PbtO2 as a monitor of cerebral oxygenation in patients with TBI [11].

The correlation between rScO2 and PbtO2 in TBI patients was also investigated by Leal-Noval et al. In their study, involving 56 patients, a weak correlation was observed between rScO2 and PbtO2 measurements. Moreover, rScO2 was shown to have poor accuracy in detecting moderate cerebral hypoxia [12], which is in agreement with the observations made by Esnault et al. [11]. Detectability improved when hypoxia reached PbtO2 values <12 mmHg [12].

Entirely different observations have been reported by Brawanski et al. Over 90% of their rScO2 readings from the frontal area and PbtO2 measurements from the white matter of the frontal lobe showed a significant correlation. An analysis of their data indicated that the PbrO2 and the rScO2 signals contained similar information despite the fact that they were obtained using completely different technologies [13]. A significant correlation between PbtO2 and rScO2 readings has also been demonstrated in patients with TBI in other research [14].

In a study comparing two types of NIRS monitors—NIRO 200, which measures the cerebral tissue oxygenation index (TOI), and INVOS 5100, which monitors rScO2, jugular bulb oxygen saturation (SjvO2) and central venous saturation (ScvO2) were also measured. Thirty one pediatric patients with congenital heart defects were examined. A significant correlation was demonstrated between the rScO2 and SjvO2 measurements, and an even higher correlation between rScO2 and ScvO2. Similarly, a high correlation between readings was observed for TOI [15].

Monitoring of cerebral oximetry using NIRS in septic shock patients allowed investigation of the relationship between rScO2 and ScvO2. rScO2 and ScvO2 values increased with the progressing stabilization of the patients’ condition and the decrease in lactic acidosis. The study demonstrated that there was a significant negative correlation between lactic acid levels and rScO2 values. On the other hand, a significant positive correlation between rScO2 and ScvO2 readings was observed at 8, 24, 48, and 72 h after admission to the ICU [16].

Many authors believe that cerebral oximetry monitoring using NIRS is an unreliable technique for monitoring oxygen metabolism in the brain tissue compared to measurements of brain tissue oxygen pressure. However, it should be noted that rScO2 and PbtO2 measure different parameters using different techniques. Moreover, PbtO2 is not acknowledged as a gold standard for monitoring oxygen metabolism in the brain. PbtO2 measurements are representative of a small area of tissue in the immediate vicinity of the probe. On the other hand, monitoring of rScO2 could prove very useful in patients in whom an invasive technique cannot or should not be used for various reasons. Both of these parameters could be monitored simultaneously to obtain a more complete picture of changes in cerebral oxygen metabolism [17]. Of note, jugular bulb oxygen saturation measurement, commonly used in TBI patients, gives a more global picture of cerebral oxygenation, and so local tissue hypoxia may go unnoticed when using this technique [18].

### 3.2. Monitoring of Cerebrovascular Autoregulation

Autoregulation of cerebral blood flow (CBF) is one of the most important mechanisms for maintaining intracranial homeostasis. It keeps CBF at a constant level in the face of changing cerebral perfusion pressure (CPP). This prevents disturbances in CBF during significant changes in arterial blood pressure. Autoregulation is extremely important in TBI patients in the context of the development of secondary injuries. As a result of impaired autoregulation, the balance between the cerebral metabolic rate of oxygen (CMRO2), cerebral blood volume (CBV) and CBF can be disturbed. This may lead to ischemic or hyperemic brain lesions, hypoxia or increased intracranial pressure. There is ample scientific evidence that abnormal autoregulation of CBF is associated with poorer treatment outcomes in patients with TBI [19,20]. Monitoring of CBF autoregulation may allow to individualize CPP targets in patients with TBI, which is associated with improved treatment outcomes [21,22].

Currently, different methods are available for assessing CBF autoregulation. The most widely used invasive techniques include measurement of intracranial pressure (ICP) and direct measurement of oxygen content in the brain tissue (parenchymal PbtO2 monitoring). Among non-invasive methods, transcranial Doppler (TCD) ultrasonography, which measures CBF velocity [23], and NIRS cerebral oximetry are commonly employed.

The values of cerebral oximetry are mainly influenced by CBF, cerebral metabolic rate of oxygen and arterial blood oxygen content. Owing to the fact that the factors affecting oxygen metabolism are relatively stable over short periods of time, NIRS can be used in place of invasive methods of bedside monitoring of changes in CBF [24,25].

Depending on the manufacturer and the technology used, NIRS devices generate various measurements, such as regional cerebral oxygen saturation (rScO2), cerebral blood flow index (rCBFi), tissue oxygenation index (TOI), and relative total tissue hemoglobin concentration (rTHb) [26].

Analyses of cerebrovascular autoregulation include computing correlations of CBF or CBV with CPP using mathematical models (e.g., COx for rScO2 or TOx for TOI) [27]. Brady et al. monitored cerebral autoregulation with a NIRS monitor in piglets using the cerebral oximetry index (COx) as an indicator of autoregulatory vascular reactivity. By correlating rScO2 values measured using NIRS with mean arterial pressure (MAP) readings, one can calculate COx. If COx is close to zero, there is no correlation between rScO2 and MAP because blood pressure is in the autoregulation range. On the other hand, COx values close to one, point to a strong correlation between cerebral oximetry and MAP, which is interpreted as impaired CBF autoregulation or as a MAP beyond the limit of autoregulation [25]. In Brady et al.’s study, COx values were compared with invasive measurements using the laser-Doppler index (LDx). Those authors demonstrated that COx displayed high sensitivity and specificity in detecting cerebrovascular autoregulation disorders caused by hypotension in piglets. The COx readings correlated with those obtained using LDx. Brady et al. showed that COx was sensitive to the loss of cerebrovascular autoregulation and could be a valuable non-invasive method for continuous monitoring of autoregulation in patients with TBI.

Monitoring of CBF autoregulation using NIRS has also been studied in patients with sepsis. NIRS readings were consistent with measurements of changes in autoregulation performed using TCD [27]. Similar results, showing the compatibility of NIRS with TCD measurements, were obtained by Zheng et al. [28].

Currently, the market offers a CBF monitoring device called c-FLOW Ornim Medical LTD device, which uses low-power ultrasound and near-infrared laser light to continuously monitor blood flow in tissue microcirculation. It tracks CBF trends, continuously measures blood pressure, and correlates the measurements to provide a real-time autoregulation index. This allows to identify the limits of cerebrovascular autoregulation and detect impairments in autoregulation [29].

### 3.3. Monitoring of Cerebral Blood Flow

In a study of non-invasive CBF measurement using NIRS and indocyanine green, Gora et al. obtained CBF values that were low compared to those obtained with conventional positron emission tomography (PET) [30]. Similarly, NIRS measurements of CBV yielded low results, which appeared to be related to contamination of the path near-infrared light travels from the emitter to the detector by extracerebral tissues. It is difficult to assess the volume and density of these tissues and their optical properties [31]. It seems that in order for NIRS to be used for the measurement of CBF and CBV in clinical settings, the contribution of extracerebral signals to NIRS readings must be further investigated [30]. Also, the influence of indocyanine green on the values of cerebral oximetry measured with NIRS does not seem to be entirely clear. Yoo et al. obtained falsely elevated cerebral oximetry readings after an intravenous bolus of indocyanine green. This marker mainly absorbs light in the near-infrared range of 600–900 nm. Therefore, one should expect that, similarly to indigo carmine or methylene blue, the use of indocyanine green would dampen cerebral oximetry readings. In Yoo et al.’s study, a several-minute increase in rScO2 co-occurred with a significantly shorter-lasting decrease in blood saturation (SpO2). The authors lean towards the theory that indocyanine green reduces the amount of light detected by NIRS devices at a wavelength of 810 nm, which is interpreted as an increase in the concentration of oxyhemoglobin and results in falsely elevated cerebral oximetry readings [32].

In contrast to these observations are the results of a study by Keller et al., in which measurements obtained by NIRS and indocyanine green were consistent with those obtained by MRI [33]. Also in the study by Milej et al. a significant correlation of the CBF values obtained with the NIRS and indocyanine green was demonstrated with the CBF measurements obtained by MRI [34].

### 3.4. Detection of Increased Intracranial Pressure

In patients with TBI, monitoring of ICP is extremely important in preventing secondary injuries. An increase in ICP is associated with a decrease in CPP, and thus a decline in CBF, which can lead, among others, to a secondary ischemic phenomenon. Moreover, elevated ICP values in patients with TBI are associated with a higher risk of death [35].

Typically, ICP is measured invasively using a probe placed inside the brain. However, attempts are being made at using NIRS cerebral oximetry measurements as a non-invasive ICP monitor.

Kampfl et al. used NIRS to monitor cerebral oximetry in two groups of patients with TBI—with ICP higher or lower than 25 mmHg. They observed significantly lower values of rScO2 in patients with increased ICP compared to the group of patients with ICP below 25 mmHg. Moreover, no differences were observed between CPP, TCD or blood gas values between these two groups. It seems that cerebral oximetry monitoring using NIRS may be of help in detecting CBF abnormalities in patients with elevated ICP [36].

Similar observations were made by Dias et al. in a study of the characteristics of plateau waves of intracranial pressure. They observed that an increase in ICP was associated with the detection of brain hypoxia by NIRS [37].

By contrast, Zuluaga et al. observed different trends in rScO2 in a pediatric population depending on the underlying disease causing intracranial hypertension. rScO2 decreased with increasing ICP in children with brain tumors and hydrocephalus, but increased when ICP was caused by intracranial hemorrhage. The authors clearly demonstrated that changes in rScO2 were not significantly related to CPP or ICP. Accordingly, NIRS does not seem to be an appropriate method for monitoring and predicting changes in ICP [38].

It should be noted that in all the studies reviewed here, authors observed changes in cerebral oximetry readings in response to an increase in ICP, thus confirming the usefulness of NIRS in detecting hypoxic episodes. However, these studies do not confirm that NIRS allows to directly diagnose changes in ICP. Nonetheless, it seems that monitoring of rScO2 in patients with TBI may be used as an auxiliary method to signal a possible increase in ICP and the need to initiate more invasive monitoring.

### 3.5. NIRS and Diffuse Correlation Spectroscopy

A newer method of monitoring CBF that uses infrared light is diffuse correlation spectroscopy (DCS), which is based on the use of the intensity fluctuations of near-infrared light.

In a study carried out on neonatal piglets with closed head injury simulating TBI, CBF was monitored with DCS and cerebral blood oxygenation using NIRS. A significant correlation of the results obtained with the DCS with the values obtained with the fluorescent microsphere technique was demonstrated. Measurements made with DCS correlated with the values of arterial oxygen saturation, mean arterial pressure and heart rate. DCS was also sensitive to changing physiological conditions such as cardiac arrest [39].

Baker et al. monitored CBF and oxidative metabolism in brain-injured adults. In the research, they used both invasive methods such as ICP monitor or cerebral microdialysis, as well as non-invasive measurement of NIRS and DCS. Conducted observations confirmed the usefulness of non-invasive methods in monitoring patients with brain injury. There were no significant correlations between the absolute values of the parameters measured with invasive and non-invasive methods. However, the NIRS and DCS measurements allowed the detection of disproportions between cerebral perfusion and oxygen metabolism during specific clinical events, which were also observed by invasive methods. The possibility of longitudinal assessment of cerebral autoregulation, based on non-invasive measurements, has also been demonstrated [40].

Due to the use of hybrid monitors, using both the NIRS and DCS techniques, it is also possible to estimate CMRO2 [41]. The combination of both monitoring techniques has also been successfully used in studies among patients undergoing elective cardiac surgery with use of circulatory arrest by measuring cerebral oximetry, CBF and CMRO2. DCS allows the identification of periods of brain hyperperfusion and hypoperfusion during circulatory arrest [42].

## 4. Limitations of the Use of Cerebral Oximetry Measurements with NIRS

Although monitoring of cerebral oximetry using NIRS has been refined over the years, it still has some limitations.

An unquestionable disadvantage of this monitoring technique is the multitude of devices available on the market, which differ in the technical aspects of making measurements, and therefore cannot be used interchangeably [43].

The results obtained with the use of NIRS are influenced by extracerebral blood flow, cerebrospinal fluid, thickness of skull bones, and myelin sheaths [44]. Interference from the lighting used in the room in which oximetry is performed, which is often overlooked in practice, is important as well. Oximetry readings can also be affected by skin pigmentation. Another problem is the falsification of measurements by myoglobin, as hemoglobin and myoglobin have similar optical properties. This may cause an overestimation of hemoglobin saturation readings [45]. Hirasawa et al. demonstrated that extracranial blood flow had an effect on cerebral oximetry readings regardless of the distance between the emitter and the detector [46].

The range of normal cerebral oximetry values is also still being discussed. The most commonly used lower and upper limits are 60% and 75%, respectively, with deviations from these baseline values as high as 10%. These limits are individually variable and depend on comorbidities [47].

In patients with TBI, there are also other issues. These patients very often have multiple wounds or postoperative sutures on the scalp, as well as subcutaneous hematomas accompanied by swelling of the soft tissues. These lesions make it impossible to properly attach NIRS electrodes, or cause disturbances in signal reception and falsify readings.

The influence of the presence of hematomas and brain edema on the absorption and scattering of near-infrared light in TBI patients has also been debated [48].

Importantly, there have been reports of measuring devices registering normal cerebral oximetry values in patients with confirmed absence of cerebral perfusion [49]. These reports seem to call into question all the other observations and the knowledge gained from them.

## 5. Conclusions

Cerebral oximetry monitoring using NIRS is increasingly employed in the therapy of TBI patients, not only to register cerebral oximetry. The greatest advantage of this method is that it enables continuous and non-invasive measurement of rScO2. Despite technical imperfections, cerebral oximetry measurements can be an important complement to other parameters monitored, providing a more holistic picture of intracranial pathologies. However, attention should be paid to the still present technical imperfections of the method, which significantly affect the obtained, often inconclusive results.

## Data Availability

Not applicable.
